# Comprehensive assessment of invalid and indeterminate results in Truenat MTB-RIF testing across sites under the national TB elimination program of India

**DOI:** 10.3389/fpubh.2023.1255756

**Published:** 2023-10-10

**Authors:** Radha Gopalaswamy, Nishant Kumar, Himanshu Vashistha, Priya Rajendran, Jyoti Kayesth, Carel Joseph Peravali, Satabdi Kashyap, Shreeparna Ghosh, Habakkuk Yumo, Moe Moore, Sridhar Anand, Ranjani Ramachandran, Umesh Alavadi, Sanjeev Saini, Sivakumar Shanmugam

**Affiliations:** ^1^ICMR-National Institute for Research in Tuberculosis, Chennai, India; ^2^Central Tuberculosis Division, Ministry of Health and Family Welfare, New Delhi, India; ^3^USAID’s Infectious Diseases Detection and Surveillance (IDDS) Project Awarded to Inner City Fund (ICF), Virginia, VA, United States; ^4^World Health Organization, New Delhi, India; ^5^United States Agency for International Development, New Delhi, India

**Keywords:** Truenat MTB-RIF testing, MTB invalid/errors, RIF indeterminate/errors, Truenat inconclusive results, RIF indeterminate, errors in Truenat testing, root cause analysis

## Abstract

**Introduction:**

Truenat MTB-RIF assay (Truenat), a nucleic acid amplification test (NAAT), is a real-time polymerase chain reaction (RT-PCR) chip-based assay that can detect *Mycobacterium tuberculosis* (*Mtb*) and rifampicin (RIF) drug resistance using portable, battery-operated devices. The National TB Elimination Program (NTEP) in India introduced this novel tool at the district and subdistrict level in 2020. This study aimed to assess the level and causes of inconclusive results (invalid results, errors, and indeterminate results) in MTB and RIF testing at NTEP sites and the root causes of these in the programmatic setting.

**Methods:**

Truenat testing data from 1,690 functional Truenat sites under the NTEP from April to June 2021 were analyzed to assess the rates of errors, invalid MTB results, and indeterminate RIF results. Following this analysis, 12 Truenat sites were selected based on site performance in Truenat testing, diversity of climatic conditions, and geographical terrain. These sites were visited to assess the root causes of their high and low rates of inconclusive results using a structured checklist.

**Results:**

A total of 327,649 Truenat tests performed for MTB and RIF testing were analyzed. The rate of invalid MTB results was 5.2% [95% confidence interval (CI): 5.11–5.26; *n* = 16,998] and the rate of errors was 2.5% (95% CI: 2.46–2.57; *n* = 8,240) in Truenat MTB chip testing. For Mtb-positive samples tested using the Truenat RIF chip for detection of RIF resistance (*n* = 40,926), the rate of indeterminate results was 15.3% (95% CI: 14.97–15.67; *n* = 6,267) and the rate of errors was 1.6% (95% CI: 1.53–1.78; *n* = 675). There was a 40.1% retesting gap for Mtb testing and a 78.2% gap for inconclusive RR results. Among the inconclusive results retested, 27.9% (95% CI: 27.23–28.66; *n* = 4,222) were Mtb-positive, and 9.2% (95% CI: 7.84–10.76; *n* = 139) were detected as RR.

**Conclusion:**

The main causes affecting Truenat testing performance include suboptimal adherence to standard operating procedures (SOPs), inadequate training, improper storage of testing kits, inadequate sputum quality, lack of quality control, and delays in the rectification of machine issues. Root cause analysis identified that strengthening of training, external quality control, and supervision could improve the rate of inconclusive results. Ensuring hands-on training of technicians for Truenat testing and retesting of samples with inconclusive results are major recommendations while planning for Truenat scale-up. The recommendations from the study were consolidated into technical guidance documents and videos and disseminated to laboratory staff working at the tiered network of TB laboratories under the NTEP in order to improve Truenat MTB-RIF testing performance.

## 1. Introduction

India remains the country with the world’s highest TB burden, with an estimated incidence of 2,950,000 (2,510,000–3,440,000) and with notification of 2.14 million TB cases in 2021; this is 18% higher than the incidence in 2020 and also represents 27% of global TB case notifications in 2021 (1). However, there was a very large gap between the number of people who fell ill with TB in 2021 and the number newly diagnosed and reported, as compared to 2019. This global gap may be attributed to both under-diagnosis and under-reporting of TB cases. To enable rapid TB testing, the WHO has recommended low- and moderate-complexity nucleic acid amplification technology (NAAT) for use in the initial diagnostic test. Among the forms of low-complexity NAAT testing available for use in peripheral settings, the WHO has recommended the use of Xpert MTB/RIF, Xpert MTB Ultra, Truenat MTB, MTB plus, MTB-RIF Dx, and TB LAMP assays (2). Of the 49 high-burden countries globally, 26 countries have reported the use of molecular tests as an initial diagnostic test for more than half of their notified cases (1). Following the WHO recommendation, India was the first country to implement Truenat MTB-RIF as an upfront molecular test, as early as 2020, under the National TB Elimination Program (NTEP) (3, 4).

Truenat MTB-RIF assay is a novel TB diagnostic tool developed by Molbio Diagnostics Pvt. Ltd., Goa, India. It is a real-time polymerase chain reaction (RT-PCR) chip-based assay that can detect *Mycobacterium tuberculosis* (*Mtb*) and rifampicin (RIF) drug resistance. With the combined advantages of affordability, simplicity in operations, diagnostic sensitivity, and portability, this micro-PCR device represents a strong candidate for wide-scale use in resource-limited settings (5).

Truenat MTB-RIF was implemented under the restrictive circumstances of COVID-19 in 2020, when TB testing was enormously reduced due to countrywide re-routing of Truenat machines for COVID-19 testing. By the end of 2021, over 1,972 Truenat machines were deployed at the district and subdistrict level under the NTEP in order to fast-track the upfront molecular testing of presumptive TB patients and identify rifampicin drug resistance in *Mtb*-positive specimens. However, the positioning of this rapid molecular TB diagnostic tool across peripheral laboratories under the NTEP in India was challenged by the rates of inconclusive Truenat results (“invalid,” “indeterminate,” or “error”). A multicenter validation study conducted previously in four reference laboratories under the NTEP showed operational advantages for the use of Truenat as an upfront molecular test (6). The diagnostic accuracy of Truenat in primary health settings and reference laboratories has been previously evaluated and found to be sufficient in a multicenter study across four different countries (7). Both studies were conducted in fewer testing centers with a controlled set-up, a limited sample size, and appropriate training of the laboratory technicians. In contrast, the present study aimed to assess the rate of inconclusive Truenat results in real-world situations and to determine their root causes. This knowledge is needed in order to optimize the testing performance of Truenat MTB-RIF, to ensure timely diagnosis, and thereby to reduce the magnitude of undiagnosed TB cases, not only in India but also in other countries that have rolled out Truenat as a molecular point-of-care tool to strengthen the TB diagnostic care cascade in national TB programs.

## 2. Materials and methods

### 2.1. Study design and settings

This was a retrospective cross-sectional study conducted at all functional Truenat sites under the NTEP in India. The study included all patients who were offered Truenat MTB-RIF testing as part of the TB diagnostic care cascade at testing sites under the NTEP across all states and union territories in India. No specific category of patients was excluded. Retrospective data on Truenat MTB-RIF testing were collected from the NTEP database and analyzed to determine the rate of invalid/indeterminate results. Truenat MTB-RIF testing uses two portable, battery-operated devices, namely, the Trueprep AUTO for nucleic acid extraction and a Truelab micro-PCR analyzer for amplification of the nucleic acids. The Truenat MTB-RIF sample pre-treatment pack contains buffers for liquefaction and lysis of the sample before the use of the Truenat AUTO for DNA elution. The lysate and elute are stored at room temperature until the completion of the tests or the end of the day, whichever is earlier. Elute DNA is subjected to amplification using a lyophilized master mix and loaded onto the Truenat MTB or RIF chip on a Truelab micro-PCR analyzer. RIF testing is conducted in the form of a reflex test for samples where MTB is detected. Re-testing for MTB or RIF is performed using the same elute or via repeat DNA extraction from the sample lysate or a fresh sample, based on the type of error or invalid result and the availability of a second sample (5). Truenat MTB testing is conducted on pulmonary and extra-pulmonary samples in the form of an upfront molecular test (2). Following the aforementioned analysis, visits were conducted to sites identified as producing high rates of invalid/indeterminate results to determine the root causes of inconclusive results; additionally, visits were conducted to a small number of sites with low rates of invalid/indeterminate results in order to understand the best practices.

### 2.2. Data collection and analysis

Retrospective Truenat testing data from 1972 functional Truenat sites for the period from April to June 2021 (2nd quarter 2021) were obtained from the NTEP and analyzed. Performance indicators for Truenat MTB-RIF testing (consisting of 45 variables) are collected from every testing site across the various states and union territories. These are submitted every quarter in the form of Microsoft Excel worksheets to CTD, India for performance monitoring. The inclusion criterion for entry into the analysis of Truenat MTB or RIF test performance was the completion of required variables as indicated for MTB or RIF testing. Truenat MTB data from 1690/1972 sites and RIF data from 1088/1972 sites were included in the study after validation, and the remaining sites were excluded from the respective analysis. Data were extracted from the individual worksheets of selected sites and curated into a single Microsoft Excel file for further analysis. The proportion of invalid and indeterminate Truenat results was analyzed and categorized according to the level of inconclusive results (Table 1).

**Table 1:** Classification of invalid MTB results and indeterminate RIF results under Truenat testing.

S. No.	% of invalid/indeterminate results	Level
1	≤5%	Very low
2	5–10%	Low
3	10–20%	Moderate
4	20–30%	High
5	More than 30%	Very high

Table 1 shows the classification of rates of invalid and indeterminate results into different levels of Truenat MTB-RIF test performance in this study. Data were analyzed using STATA version 16.0 and the proportions of invalid/indeterminate results obtained in Truenat testing at selected sites were determined based on descriptive statistics.

### 2.3. Root cause analysis

**Figure 1:**
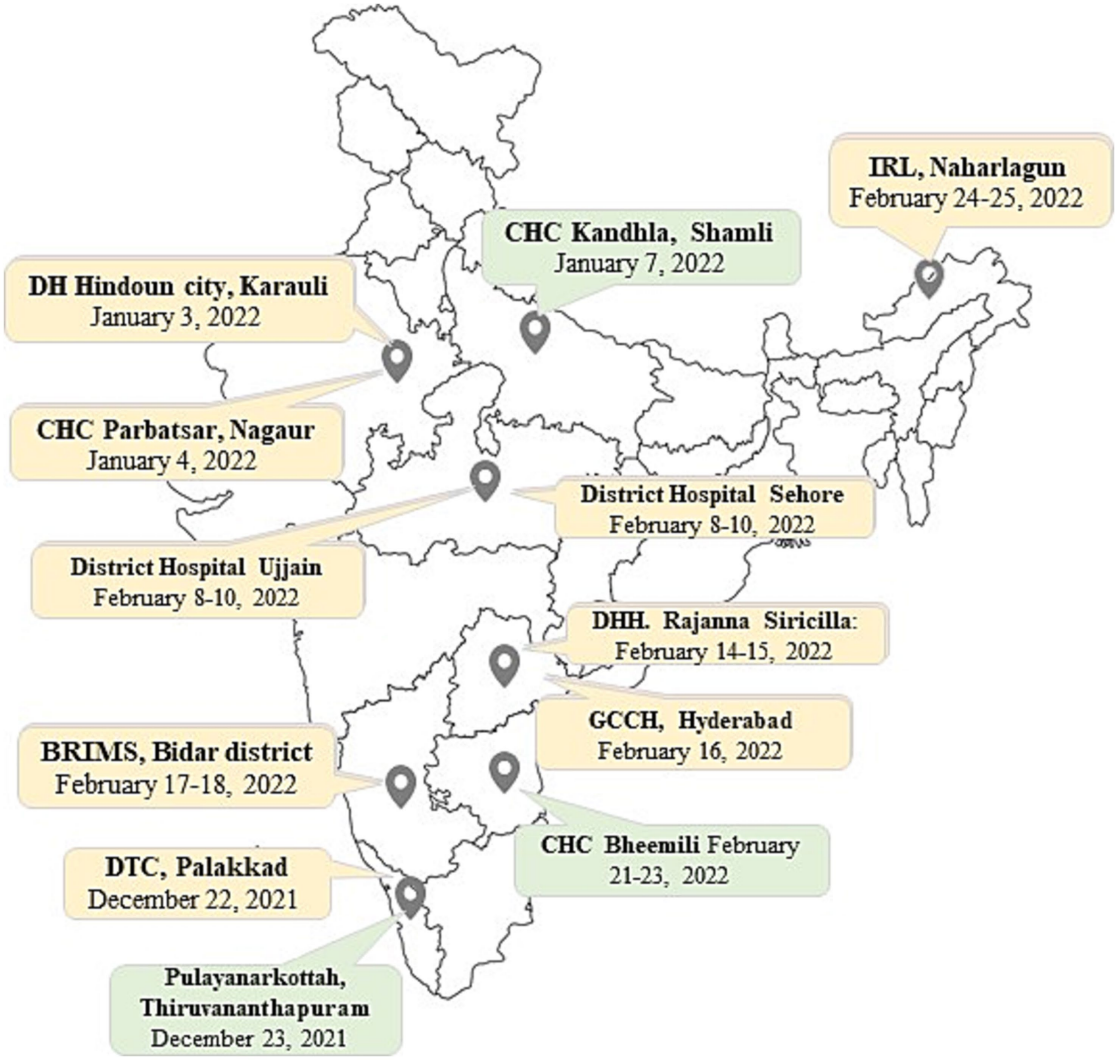
Figure shows the 12 Truenat sites visited for root cause analysis on a map of India. Three sites, marked in green, showed good performance for that quarter while nine sites, marked in yellow, showed poor performance during the study period.

Of 1,690 sites analyzed, 81 met the inclusion criteria for site visits to determine the root causes of inconclusive results, and the remainder were excluded from this component of the study. The inclusion criteria were organized into five categories (A–E) depending on the rates of invalid/error results for MTB and indeterminate/error results for RIF. Additionally, for every criterion, specific exclusions were made based on the number of MTB or RIF tests performed per quarter (Table 2). Of these 81 sites, 12 were selected for root cause analysis visits (Figure 1). Site selection was performed based on a combination of criteria, including the rate of inconclusive Truenat results (Tables 1, 2) and several site parameters, including laboratory workload, geographical distribution, weather conditions, and the location of some sites in remote areas with sample transportation challenges.

**Table 2:** Site selection criteria for root cause analysis visits.

Sites selection criteria	Invalid MTB rate	Indeterminate RIF rate	MTB tests conducted per day	Exclusion criteria	Reason	No. of sites	No. of sites visited
Criterion A	≤5%	≤5%	5–8 or more than 8 tests/day	<300 MTB tests/quarter (qtr); <60 RIF tests/qtr	High test rate + good performance	18	2
Criterion B	≤5%	≤5%	≤1 tests/day or 2–4 tests/day	<100 MTB tests/qtr; <60 RIF tests/qtr	Low test rate + good performance	20	1
Criterion C	>10%	>10%	5–8 or more than 8 tests/day	<300 MTB tests/qtr; <60 RIF tests/qtr	High test rate + high rates of invalid MTB and indeterminate RIF results	18	5
Criterion D	>10%	>10%	≤1 tests/day or 2–4 tests/ day	<300 MTB tests/qtr; <60 RIF tests/qtr	Low-to-medium test rate + high rates of invalid MTB and indeterminate RIF results	6	2
Criterion E	<5%	>20%	All groups considered	<300 MTB tests/qtr; <60 RIF tests/qtr	Any test rate + low rates of invalid MTB results + high rates of indeterminate RIF results	19	2

Table 2 shows site selection for root cause analysis based on rates of invalid MTB and indeterminate RIF results in Truenat MTB-RIF testing. Among the sites meeting the criteria, further selections were made based on the numbers of MTB and/or RIF tests per day.

Site visits were conducted to the selected Truenat sites (Figure 1) to assess the underlying reasons for inconclusive Truenat results. Representatives from the Central TB Division (CTD) and/or the Indian Council of Medical Research–National Institute for Research in Tuberculosis (ICMR-NIRT) Infectious Disease Detection and Surveillance (IDDS) team jointly conducted the root cause analysis visits between December 2021 and February 2022. Four to five team members participated in every visit; they included program managers, microbiologist(s) and/or biotechnologist(s), and implementing partners from different tiers of the TB diagnostic care cascade in India. During the visits, the laboratory and other work practices of the staff performing Truenat tests were observed, and the findings were documented after focus group discussions with laboratory technicians (LTs), senior TB laboratory supervisors (STLSs), and District TB Officer (DTO) of each site using a comprehensive checklist (Supplementary Table S1). The checklist included site characteristics such as laboratory infrastructures, workload capacity, and turnaround time, as well as qualitative questions covering key areas of operational procedures related to Truenat testing, such as training and competency assessment, availability of SOPs, storage conditions of reagents and kits, quality assurance systems, equipment upkeep, sample transportation, technical procedures, and recording and reporting practices. The checklist included questions aiming to identify the root causes of high rates of invalid and/or indeterminate results at individual sites. Root cause analysis were performed based on the answers obtained from the site visits (Figure 1).

Qualitative data analysis involved the identification, examination, and interpretation of the information collected in order to identify emergent patterns and common themes related to the occurrence of invalid/indeterminate Truenat results at the selected sites.

## 3. Results

### 3.1. Truenat MTB testing

Data reported between April and June 2021 (3 months) by 1,690 Truenat sites distributed across 32 states and union territories (UTs) were analyzed. Of the 45 Truenat-related variables reported (which included both Truenat test performance and patient characteristics, with patients stratified into presumptive, presumptive drug-resistant, people living with HIV (PL-HIV), pediatric, extra pulmonary (EP), and previously treated TB cases), only 14 variables relevant to the study objectives (i.e., only those pertaining to Truenat test performance) were shortlisted for further analysis (Tables 3, 4). A total of 327,649 Truenat MTB and RIF resistance (RR) tests were conducted for the diagnosis of TB among presumptive TB patients and for the detection of RR among confirmed TB patients. Overall, 22.1% of the samples (72,504/327,649; 95% confidence interval (CI): 21.99–22.27) were identified as positive for *Mtb*. Among all TB patients/samples tested, the proportions of invalid MTB results and errors were 5.2% (*n* = 16,998; 95% CI: 5.11–5.26) and 2.5% (*n* = 8,240; 95% CI: 2.46–2.57), respectively, corresponding to a total rate of inconclusive Truenat results of 7.7%. A total of 40.1% (*n* = 10,124) of the inconclusive results were not retested. Among retested presumptive TB patients/samples (*n* = 15,114) with initially inconclusive MTB results, 27.9% (*n* = 4,222; 95% CI: 27.23–28.66) were detected as positive for *Mtb* (Table 3). Rates of invalid MTB results varied between states/UTs, from 0% in Manipur and Puducherry to 12.6% in Andhra Pradesh (Supplementary Figures S1A,B).

**Table 3:** Analysis of Truenat MTB testing.

Variable	*N*	% (95% CI)
Total no. of sites with Truenat results	1,690	–
Total number of MTB tests performed using Truenat MTB chip	327,649	–
Total number of MTB “not detected” results (MTB-)	72,504	22.1 (21.99–22.27)
Total number of MTB “detected” results (MTB+)	2,32,328	70.9 (70.75–71.06)
Total number of test showing an “invalid” result (MTB invalid)	16,998	5.2 (5.11–5.26)
Total number of errors (in cases where MTB chips were used)	8,240	2.5 (2.46–2.57)
Number of repeat tests for TB diagnosis out of total errors / invalid results	15,114	59.9 (59.28–60.49)
Number of MTB “detected” results out of errors/invalid results	4,222	27.9 (27.23–28.66)

*N*, number; CI, confidence interval.

Table 3 shows an analysis of Truenat MTB testing based on estimation of certain critical variables, including MTB “not detected” results, MTB “detected” results, invalid results/errors, retesting of inconclusive results, and MTB detection upon retesting.

**Table 4:** Analysis of Truenat RIF testing.

Variable	*N*	% (95% CI)
Total no. of sites with Truenat RIF results	1,088	–
Total number of RIF tests performed using Truenat RIF chip	40,926	–
Total number of RIF “not detected” results (RIF-)	2,006	4.9 (4.70–5.12)
Total number of RIF resistance “detected” results (RIF+)	31,978	78.0 (77.74–78.54)
Total number of tests showing indeterminate results	6,267	15.3 (14.97–15.67)
Total number of errors (in cases where RIF chips were used)	675	1.6 (1.53–1.78)
Number of repeat tests for rifampicin resistance out of errors/indeterminate results	1,514 (*n* = 1,047)*	21.8 (20.86–22.80)
Number of tests positive for rifampicin resistance out of errors/indeterminates	139 (*n* = 1,047)*	90.2 (7.84–10.76)

*N* = number; CI = confidence interval. ^*^Outliers for RIF resistance: errors/indeterminate results were not considered due to data discrepancy.

Table 4 shows an analysis of Truenat RIF testing based on estimation of certain critical variables, including RIF “resistance not detected” results, RIF “resistance detected” results, indeterminate results/errors, and retesting of inconclusive results, and RIF status upon retesting.

### 3.2. Truenat RIF testing results

Among the 1,690 Truenat sites, 602 sites were excluded from the RIF testing analysis due to observed discrepancies in the reported data. These 602 sites had entered erroneous data that could not be validated into the Microsoft Excel sheets used for Truenat data collection. The types of discrepancies included: (i) mismatch in the data in terms of number of RIF tests as compared to total cases of MTB detected; (ii) data entered as zero (0) in the Excel sheets; (iii) identical entries in all variables (data duplicity); and (iv) a lack of data on RIF variables for some Truenat sites due to a lack of RIF chips and hence these sites not having performed Truenat RIF testing. Hence, a total of 1,088 sites were included in the RIF testing analysis; across these sites, 53.3% of samples having tested positive for *Mtb* (40,926/76,726, including repeats) were further evaluated via RIF testing. RR was detected in 4.9% (2,006/40,926; 95% CI: 4.70–5.12). The proportions of indeterminate results and errors in RR testing were 15.3% (*n* = 6,267;95% CI: 14.97–15.67) and 1.6% (*n* = 675;95% CI:1.53–1.78), respectively, corresponding to an overall rate of inconclusive results of 16.9%. Among the RR inconclusive results, 21.8% of patients (1,514/6,942) were retested for RR, and in 9.2% of these cases (139/1,514; 95% CI: 7.84–10.76), RR was detected (Table 4).

The rates of inconclusive results in RIF testing varied between states, from 4% in Jharkhand to 100% in Manipur and Puducherry states. However, Puducherry and Manipur reported only one and two samples as having been tested for RIF, respectively. As a very small number of samples were tested, proportions of inconclusive results in these states need to be considered as outliers (Supplementary Figures S2A,B).

### 3.3. Observations

In our root cause analysis, we defined uniform baselines of ≤5% invalid/indeterminate results for good performance in MTB-RIF testing and > 10% for poor performance. The rates of inconclusive results in Truenat *Mtb* testing were very low (<1%) in Manipur, Puducherry, Goa, and Sikkim states and high (>10%) in Uttarakhand and Rajasthan states. Likewise, the rates of inconclusive Truenat RR results were relatively low (<10%) in states like Jharkhand and Andhra Pradesh and very high (50%) in Nagaland, Sikkim, Manipur, and Puducherry states. There was wide variation in the proportions of inconclusive Truenat *Mtb* and RR testing results, which could have been influenced by the small number of tests conducted in some states and higher levels of testing in others (Supplementary Figures S1A, S2A). After qualitative analysis of the observations documented in the checklist during site visits, the major reasons, identified individually, for inconclusive *MTB* and RIF testing results were as follows.

**Table:** 

Reasons for high rates of invalid results in MTB testing	Reasons for high rates of indeterminate results in RIF testing
1. Issues related to device maintenance: heater plate malfunctioning, chip carrier tray and pinion not functioning, valve V1 damage, damage to switchboard light-emitting diode (LED), motherboard failure.	1. Testing on paucibacillary load samples (EPTB, UDST >8 weeks).
2. Faulty reagent pack supply at some sites: leakage, manufacturing defect, not able to connect properly.	2. Issues with laboratory technician (LT) proficiency.
3. Delay in rectification of machine faults.	3. Non-compliance with standard operating procedures.
4. Validation of machine and quality control testing not performed after device shifting.	4. Improper storage conditions for reagents and chips; melting of wax inside the reaction well due to high temperatures.
5. Lack of monitoring of sample quality by technical staff (LTs).	5. Non-adherence to TB diagnostic algorithm.
6. Improper storage conditions for reagents and chips; melting of wax inside the reaction well due to high temperature ranges.	6. Lack of hands-on training and pipette calibration issues.

## 4. Discussion

The analysis of 327,649 presumptive TB patients tested in a 3-month period at 1,690 Truenat sites distributed across 89% of the states and UTs of India (*n* = 32) indicated that 22.1% of presumptive TB cases were *Mtb*-positive. This rate was higher than the 14.1% reported by Adam Penn-Nicholson et al. in a multi-country study (7) or the 13.0% reported by Abyot Meaza et al. in Ethiopia (8). The reason for the higher rate of *Mtb*-positives may be the testing of samples from smear-positive TB patients for universal drug susceptibility testing (UDST) in addition to presumptive TB patients at a small number of the sites. In India, a total of 2,197,757 Truenat MTB tests were performed in 2021, with a positivity rate of 20.5% (9). Our study indicated an MTB positivity rate of 22.1% during the period April to June 2022 in Truenat MTB-RIF testing.

In programmatic settings in India, a second sample from patients determined to be positive for Mtb should be sent to a culture and drug susceptibility testing (DST) facility for first−/second-line probe assay (FL/SL LPA), followed by liquid culture DST for all drug-resistant samples detected by LPA (10). In 2021, 29,84,636 presumptive TB patients were tested via NAAT, and Mtb was detected in 8,73,725. However, only 3,28,715 samples were tested via FL-LPA, and 46,733 resistant TB cases were identified. SL-DST was performed for 14,886 samples, but there are no reports on the numbers of samples tested by DST facilities for newer drugs (9). These numbers indicate a gap between initial and follow-on resistance testing and reinforce the need for efficient upfront molecular testing. The Truenat testing system has great aspirations to fill in this gap in the diagnostic pipeline. Under the DR-TB regimen, all patients except those whose samples indicate isoniazid resistance are started on an MDR/RR-TB regimen, and early identification of rifampicin resistance status could help with initiation of this treatment regimen.

Hands-on training is important for Truenat testing as, unlike GeneXpert, the process involves additional manual steps for DNA extraction and requires the handling of pipettes. Under a program fo POC molecular diagnosis, such as Truenat, it becomes highly critical that the lab technicians are well trained in the various methods and processes involved in the technique, such as micropipette handling and knowledge of workstation cleanliness, disinfection techniques, and proper operation of instruments. Even subtle factors in handling micropipettes, such as immersion angle, immersion depth, ergonomic practices, maintenance, and periodic servicing, are highly critical for good laboratory practice, which would help to consistently produce a higher rate of concordant molecular diagnosis of TB (11). Continuous monitoring of the competency of LTs by STLS/supervisory staff would help to resolve issues at the earliest possible stage. Although training of trainers was conducted by the NTEP in March 2020, cascade training of IRLs and district staff could not be carried out due to the COVID-19 pandemic. Virtual training sessions were conducted for the staff, which were not as effective as in-person, hands-on training. Considering this gap due to unforeseen situations, it has recently been suggested by the NTEP that all the IRLs conduct in-person training of staff.

Inconclusive results on the Truenat MTB-RIF occur for multifactorial reasons, all of which need to be addressed, and gaps between policies and testing efficiency must be identified (12, 13). In this study, the rates of invalid Truenat MTB/RIF results were lower (5.2%) compared to the 11.6% reported in Ethiopia (8), while the rate of inconclusive results (6.7%) corroborated those of a previous study in India (6). Despite the observed low proportions of inconclusive RR results (16.9%), these unsuccessful outcomes could lead to meaningful missed opportunities in DR-TB case detection. The lack of retesting of inconclusive RR results indicates the potential negative impact that the inconclusive Truenat results may have in DR-TB case detection. The study identified a testing gap of 41.1 and 78.2% for MTB and RIF testing, indicating a lack of knowledge of the current Truenat testing algorithm in India recommending retesting in the case of inconclusive results. The study indicates a need to draw up a plan for rigorous training emphasizing iteration of Truenat MTB-RIF testing to obtain a conclusive result. Samples eliciting inconclusive Truenat MTB-RIF results are recommended to be tested at the same testing site using the same DNA eluate, and if the outcome is not resolved, then a fresh specimen can be used (5, 10). A multicenter study conducted by FIND (the Foundation for Innovative New Diagnostics) showed that retesting of samples reduces inconclusive results from 6.2 to 1.7% for the Truenat MTB test (7). The performance indicator recommended for the rate of inconclusive results is <3% for MTB testing (5). With rigorous training, competency assessment, and quality monitoring by the Central TB Division (Ministry of Health and Family Welfare), with the respective NRL and IRL, the nation could achieve the expected level of technical proficiency. Additionally, the rate of indeterminate RIF results has been reported to be high for Truenat MTB testing, even after retesting, particularly when the bacillary load was low (7). The quality of the sample is also critical for proper diagnosis when using extrapulmonary specimens. Paucibacillary status and the presence of potential inhibitors of PCR in non-sputum samples pose additional challenges for the diagnosis of TB using molecular techniques, such as by the Truenat method (14, 15). Previous studies on Xpert MTB/RIF have also indicated similar issues with sample processing and operator error, indicating technical, machine maintenance, and operational issues leading to higher rates of inconclusive results (16, 17).

The MTB test results provide a cycle threshold (cT) value for the target gene and an estimated bacterial load in the form of colony-forming units per ml (CFU/ml) (5). Based on our study, we recommend the inclusion of cT and CFU/ml as additional information in the performance indicators for Truenat MTB testing. Low bacillary load samples and their corresponding RIF status could be validated, and this would improve the overall implementation and effectiveness of the Truenat MTB/RIF test.

The root cause analysis visits conducted at 12 selected sites showed that the main causes of inconclusive results included faults related to machine maintenance, inadequate sample quality, and inadequate quality control. The Guidelines for Truenat testing recommend performing a quality control test with negative and positive control samples routinely and on occasions when the storage temperature of the Truenat chips falls outside the recommended temperature range of 2–30°C (5). These issues need to be addressed appropriately to ensure the optimal outcome of Truenat testing at the peripheral level. Effective external quality control and formative supervision could help with this objective.

The main observations of our study include:

- The key observations made at the sites with <5% invalid MTB results and indeterminate RIF results were good pipetting practices, periodic machine maintenance, clean and clutter-free work surfaces subjected to regular disinfection, proper storage conditions for chips, timely processing of samples without any backlog, and proper liquification of samples.
- Errors in MTB and RIF testing were predominantly due to the pipette not being changed every 6 months as recommended by the manufacturer, as well as machine-related issues.
- Delays in rectification of machine faults by the service engineer contributed to higher rates of inconclusive results in Truenat assay.

However, we also observed that a well-performing site can also perform poorly if a well-trained technician is replaced with an untrained one, and vice versa. Supervision and monitoring of performance with proper recording of the reasons for an invalid or indeterminate test result, including error type, is essential. Reinforcement of retesting should be conducted across various sites to ensure a reduction in inconclusive results.

The limitations of this study are related to missing information from the retrospective data collected from the Truenat sites. The lack of complete data on MTB testing or the mismatched or missing data observed in the case of RIF testing may have led to the exclusion of a well-performing or poorly performing site, causing some degree of bias in our analysis. However, any recall bias during the site visit was mitigated by triangulating data from different sources (Truenat machines, physical laboratory records at the sites, and the Truenat indicators submitted to the CTD through designated reference laboratories under the NTEP). Another limitation was the restriction to 12 site visits from among 81 sites listed initially under various criteria. However, owing to travel restrictions due to COVID, the study personnel limited their visits to sites chosen based on their testing rates, performance, geographical location, climatic conditions, and remoteness to ensure that all factors were covered in the root cause analysis.

## 5. Conclusion

The present study has provided the desired knowledge on the magnitude and the potential negative impact of inconclusive Truenat results in DR-TB case detection and performed root cause analysis to identify appropriate solutions to optimize Truenat MTB-RIF testing. Our major findings substantiate the significance of retesting samples that produce inconclusive Truenat results and the value of this method in rapid DR-TB treatment initiation. Our key recommendations for Truenat MTB-RIF testing under the National TB Elimination Program of India, as well as other countries that have deployed Truenat MTB-RIF assay in their diagnostic algorithm, in order to optimize the implementation and outcomes of Truenat assay, include:

- Dedicated staff;
- Regular training of laboratory technicians;
- Good documentation practice in Truenat MTB-RIF reporting;
- Meticulous retesting to obtain a conclusive result;
- Sample quality monitoring; proper storage and quality control;
- Recording of pipette calibration and machine maintenance at all sites.

Our study showed that well-performing sites with high sample loads had met most of these requirements, which enabled good performance in Truenat MTB-RIF testing. The recommendations highlighted above were consolidated into guidance documents and videos and disseminated nationwide by the CTD, Ministry of Health and Family Welfare, Government of India, in order to improve Truenat MTB-RIF testing. The videos emphasized a set of Good Laboratory Practices (GLPs) and “Do’s and Don’ts” in Truenat MTB-RIF testing (18). Guidance on the roles and responsibilities of the program managers, laboratory supervisory staff, and technical staff to help optimize Truenat testing at NTEP sites was prepared (18). This guidance document included key factors, including programmatic aspects; technical aspects; aspects of recording and reporting; quality assurance; and instrument maintenance for efficient implementation, supervision, and performance of Truenat MTB-RIF assay.

## Data availability statement

The raw data supporting the conclusions of this article will be made available by the authors, without undue reservation.

## Ethics statement

The studies involving humans were approved by the ICMR-National Institute for Research in Tuberculosis (NIRT), Chennai and ICF Incorporated LLC Institutional Review Board (IRB). The studies were conducted in accordance with the local legislation and institutional requirements. The human samples used in this study were acquired from a by- product of routine care or industry. Written informed consent for participation was not required from the participants or the participants’ legal guardians/next of kin in accordance with the national legislation and institutional requirements.

## Author contributions

RG: Conceptualization, Writing – original draft. NK: Conceptualization, Writing – review & editing. HV: Conceptualization, Writing – original draft. PR: Writing – review & editing. JK: Writing – review & editing. CP: Writing – review & editing. SK: Writing – review & editing. SG: Writing – review & editing. HY: Writing – review & editing. MM: Writing – review & editing. SA: Writing – review & editing. RR: Writing – review & editing. UA: Writing – review & editing. SaS: Conceptualization, Supervision, Writing – review & editing. SiS: Conceptualization, Supervision, Writing – review & editing.
