# Supplementary material for: Comprehensive assessment of invalid and indeterminate results in Truenat MTB-RIF testing across sites under the national TB elimination program of India
Source: Front Public Health. 2023 Oct 10;11:1255756. doi: 10.3389/fpubh.2023.1255756 (PMC10598606; doi:10.3389/fpubh.2023.1255756)
Supplement: Supplementary file 1 [file Data_Sheet_1.docx]

Supplementary Material

# Supplementary Data

Supplementary figures:

Figure S1a: State-wise distribution of Invalid Rates in *Mtb* performing less than 5000 tests per quarter


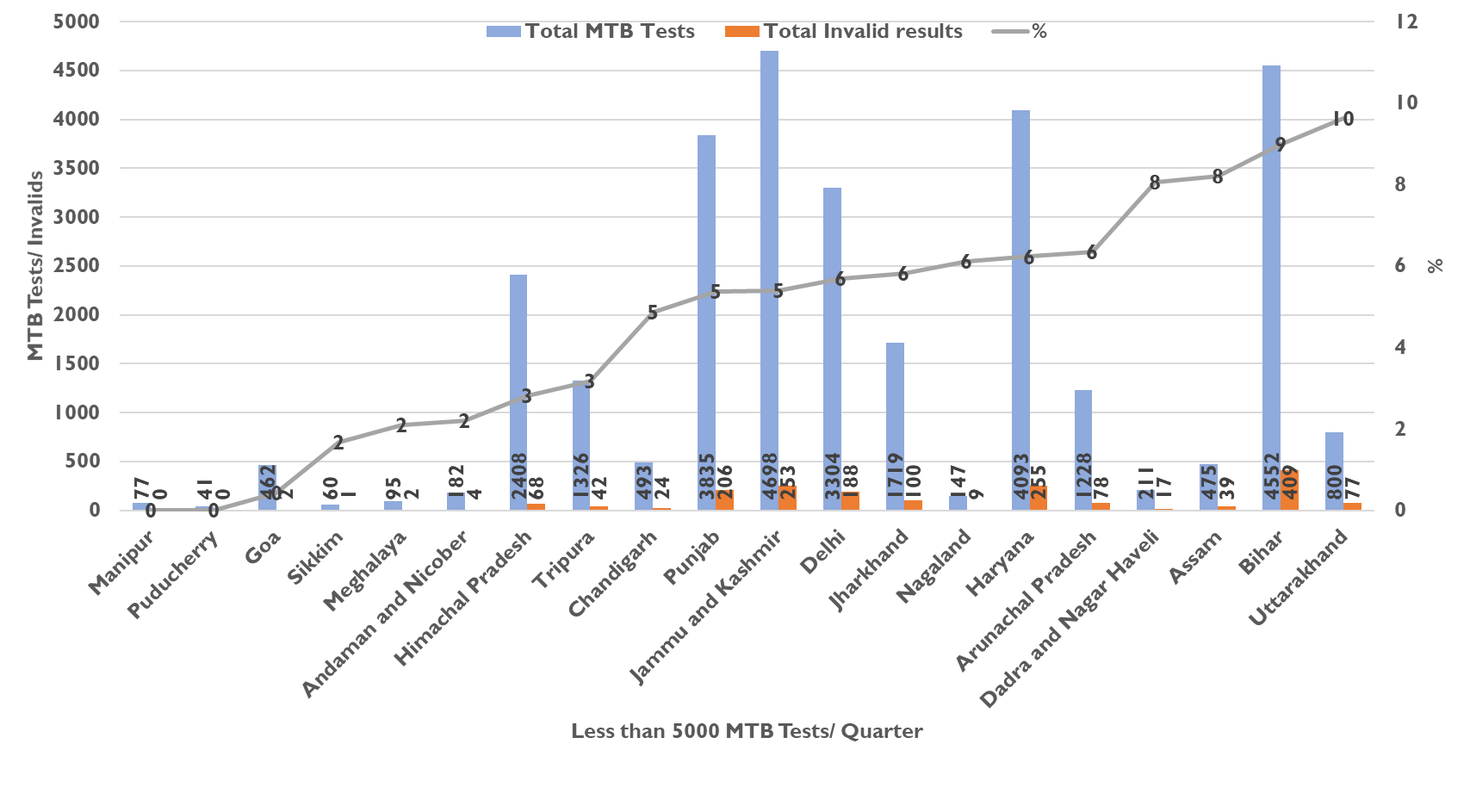


The graph shows state-wise distribution of Invalid Rates in *Mtb* performing less than 5000 tests per quarter. Blue bar represents total MTB tested and orange bar represents the total invalid results. The line graph shows the percentage of invalids among the total MTB tested.

Figure S1b: State-wise distribution of Invalid Rates in *Mtb* performing more than 5000 tests per quarter


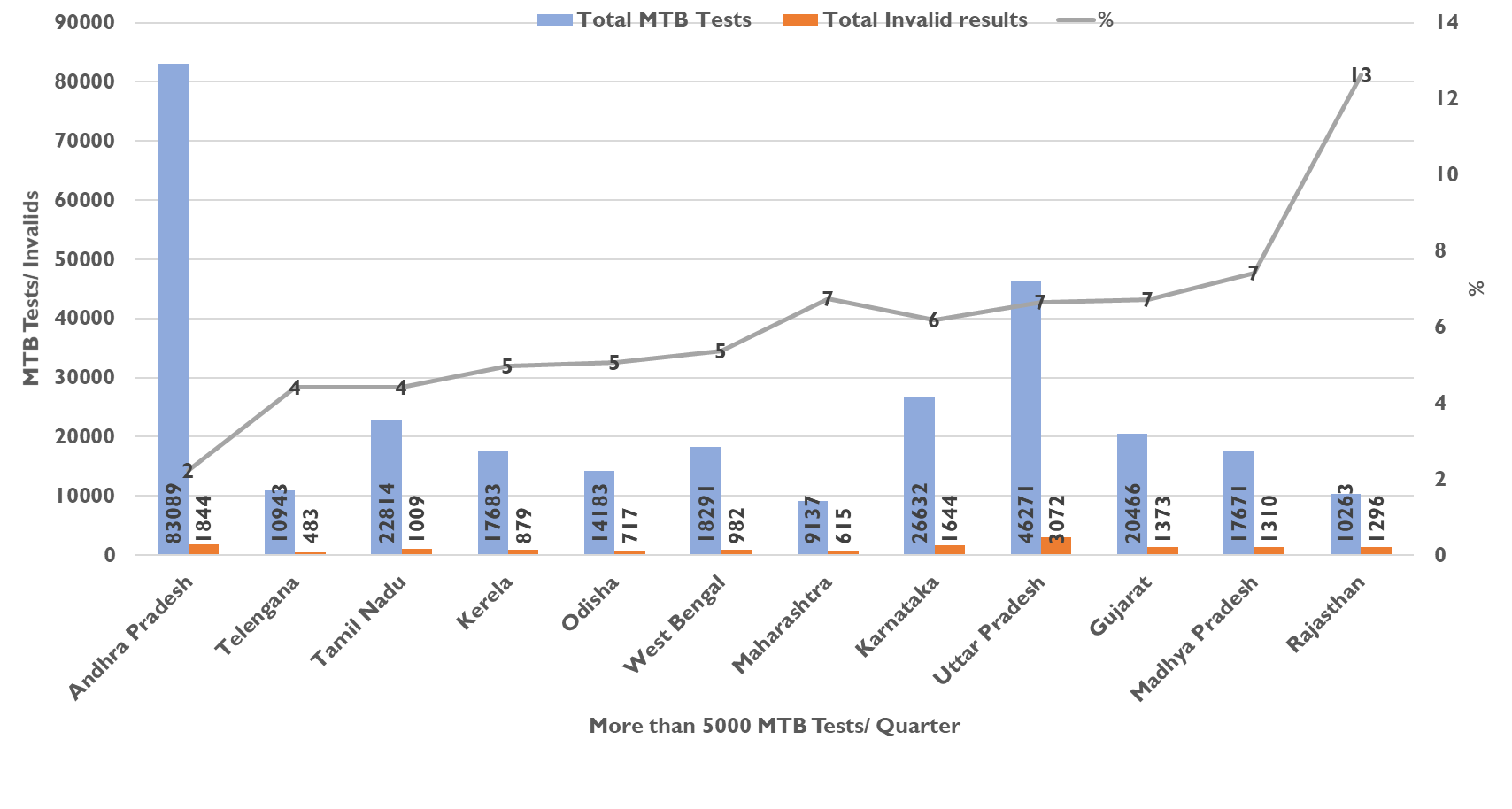


The graph shows state-wise distribution of Invalid Rates in *Mtb* performing more than 5000 tests per quarter. Blue bar represents total MTB tested and orange bar represents the total invalid results. The line graph shows the percentage of invalids among the total MTB tested.

Figure S2a: State wise distribution of Indeterminate Rates in Rifampicin performing less than 1000 tests per quarter


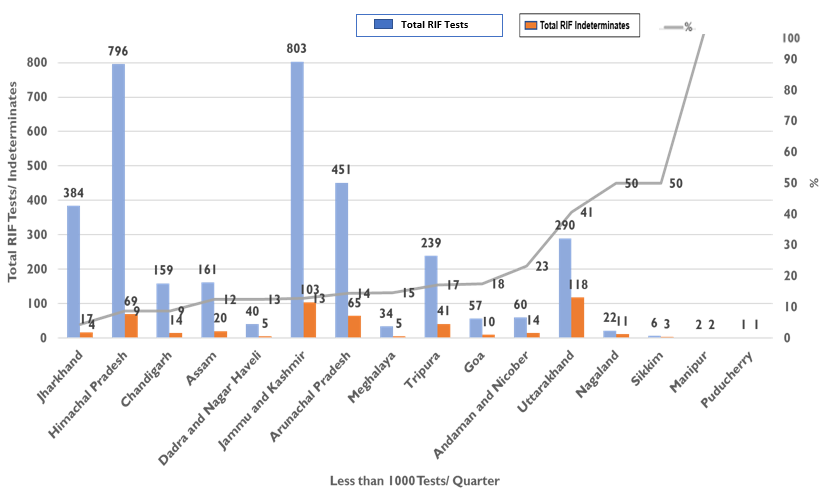


The graph shows state-wise distribution of Indeterminate Rates in Rifampicin testing performing less than 5000 tests per quarter. Blue bar represents total RIF tested and orange bar represents the total indeterminate results. The line graph shows the percentage of indeterminates among the RIF tested.

Figure S2b: State wise distribution of Indeterminate Rates in Rifampicin performing more than 1000 tests per quarter

#
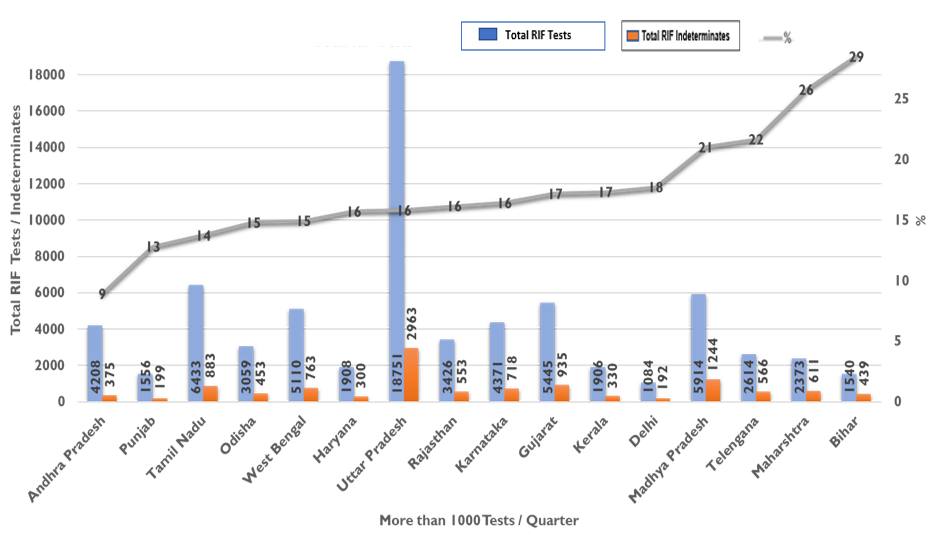


The graph shows state-wise distribution of Indeterminate Rates in Rifampicin testing performing more than 5000 tests per quarter. Blue bar represents total RIF tested and orange bar represents the total indeterminate results. The line graph shows the percentage of indeterminates among the RIF tested.
