# Supplementary material for: Comprehensive assessment of invalid and indeterminate results in Truenat MTB-RIF testing across sites under the national TB elimination program of India
Source: Front Public Health. 2023 Oct 10;11:1255756. doi: 10.3389/fpubh.2023.1255756 (PMC10598606; doi:10.3389/fpubh.2023.1255756)
Supplement: Supplementary file 2 [file Data_Sheet_2.docx]

Supplementary Material

# Supplementary Data

**1.1** **Supplementary table 1**

|  | **Infectious Diseases Detection and Surveillance (IDDS)** | | | | |
| --- | --- | --- | --- | --- | --- |
|  | **Site visit Checklist for Truenat Invalid/Indeterminate study** | | | | |
|  | **Details of Truenat Site:** | | | | |
|  | **Name of Linked Reference lab:** | | | | |
|  | **Location of site (City/town, District, State):** | | | | |
|  | **Laboratory Level: National ( ), Intermediate ( ), Peripheral ( )** | | | | |
|  | **Contact person Name, designation & email Id:** | | | | |
|  | **Specimens collected / month in numbers:** | | | | |
|  | **Referred specimens received per month:** | | | | |
|  | **Truenat Instrument (s) details:** | | | | |
|  | **2 Quarter data** | **3 Quarter data** | | | |
|  | **Truenat Invalid rate in MTB:** | **Truenat Invalid rate in MTB:** | | | |
|  | **Truenat Error Rate in MTB:** | **Truenat Error Rate in MTB:** | | | |
|  | **Truenat Indeterminate Rate in RIF DR:** | **Truenat Indeterminate Rate in RIF DR:** | | | |
|  | **Truenat Error rate in RIF DR:** | **Truenat Error rate in RIF DR:** | | | |
| **S#** | **Observations:** | **Yes** | **No** | **Partial** | **Comments** |
| **A** | **Training and competency assessment** | | | | |
| 1 | Are the following national guidelines, SOP's and plans accessible at the testing site:  • NTEP TB diagnostic algorithm that includes the use of Truenat TB testing? • SOP for Truenat TB test? |  |  |  |  |
| 2 | Has a staff member (part-time or full-time) been appointed in the laboratory to oversee the Truenat TB testing? |  |  |  |  |
| 3 | • On-site training and competency assessments? |  |  |  |  |
| 4 | • Quality assurance (proficiency testing, monitoring indicators etc.)? |  |  |  |  |
| 5 | • Projected on-going costs related to Truenat TB testing (staff, reagents, consumables, etc.)? |  |  |  |  |
| 6 | Has the site provided Truenat TB training for on-site healthcare workers on: (Fill the competency assessment part of the checklist) |  |  |  |  |
| 7 | • TB/DR-TB Diagnostic algorithm? |  |  |  |  |
| 8 | • Sample rejection criteria? |  |  |  |  |
| 10 | Is the technician performing the test trained as per the NTEP guidelines? |  |  |  |  |
| 11 | The training is performed by whom? (training at IRL or onsite by Molbio) |  |  |  |  |
| 12 | Have all healthcare workers in sites that will be referring specimens to this site been trained on all of the above components? |  |  |  |  |
| 13 | Are there any additional training needs for lab staff? |  |  |  |  |
| B. | **Site Infrastructure** | | | | |
| 1 | Does the laboratory have a lockable door and secure windows |  |  |  |  |
| 2 | Is there adequate space for receiving and processing specimens? |  |  |  |  |
| 3 | Is there adequate amount and quality of bench space for the Trueprep and Truelab instruments and ancillary equipment (s)?  • flat, stable surfaces  • vibration-free or away from instruments that cause electromagnetic interference  • out of direct sunlight  • away from to any radiating or heating apparatus |  |  |  |  |
| 4 | Are the Truenat TB instruments placed correctly in the laboratory? |  |  |  |  |
| 5 | Are Truenat TB workstations clean, free of clutter, and organized for efficient operation? |  |  |  |  |
| 6 | Does the testing site provide sufficient ventilation and biosafety for Truenat TB testing procedures? |  |  |  |  |
| 7 | Does the testing site ensure an optimal working temperature (15°C to 40°C) and environment (humidity [10-80%], dust-free) for the Truelab instruments? |  |  |  |  |
| 8 | Is available electricity adequate for Truenat TB testing and battery charging? |  |  |  |  |
| 9 | Are there 3 available sockets on the lab bench for the Truenat instruments? |  |  |  |  |
| 10 | If needed, is a power backup or solar powered generator available for charging of Truelab instruments? |  |  |  |  |
| 11 | Is there sufficient, secured, and organized storage space for chips  (2°C–40°C) and Truenat TB reagents / kits (2°C–40°C) ? |  |  |  |  |
| 12 | Is there documented monitoring and review of environmental temperatures at the testing and storage areas? |  |  |  |  |
| 13 | Does the laboratory have a refrigerator for storing sputum samples? |  |  |  |  |
| 14 | Does the testing site use appropriate disinfectants and are they prepared correctly? |  |  |  |  |
| 15 | Is suitable personal protective equipment (PPE) provided at the testing site and are staff trained in its correct use? |  |  |  |  |
| 16 | Is specimen processing area well ventilated and well lit? |  |  |  |  |
| 17 | Does the testing site segregate waste and dispose of it as per BMW SOP's or national regulations or guidelines? |  |  |  |  |
| 18 | Does the testing site have adequate capacity for safely and properly disposing of the anticipated significant amount of plastic waste generated by Truenat testing? |  |  |  |  |
| C | **Equipment, service and maintenance** | | | | |
| 1 | Is a routine maintenance log available indicating daily, weekly, and monthly tasks? |  |  |  |  |
| 2 | Is a service contract in place to provide comprehensive service and maintenance? |  |  |  |  |
| D | **Supply chain** | | | | |
| 1 | Does the staff know about the estimation of Truenat consumables and monitor inventory (physical count)? |  |  |  |  |
| 2 | Are Truenat TB testing supplies available at the testing site, in-date, labeled with receive date, organized and stored at recommended storage conditions? |  |  |  |  |
| 3 | Is quality control testing (QC) performed on new lots of Truenat TB reagents prior to their use for testing patient samples to ensure that they perform as expected? |  |  |  |  |
| E | **Sample transportation (Discussion)** | | | | |
| 1 | Is there an established sample transportation system from clinical sites to the Truenat TB testing laboratory? if yes, describe the current system, its adequacy, efficiency and coverage |  |  |  |  |
| 2 | Is a sample referral system in place for additional testing for TB samples at referral laboratories (e.g., second-line DST)? If yes, describe the current system, its adequacy, efficiency and coverage |  |  |  |  |
| F | **Procedures** | | | | |
| 1 | Are all of the needed standardized documents, records and forms related to Truenat TB testing readily accessible to all staff? |  |  |  |  |
| 2 | • Truenat TB test requisition form/ Form 15 |  |  |  |  |
| 3 | • Laboratory register |  |  |  |  |
| 4 | • Truenat TB instrument maintenance log |  |  |  |  |
| 5 | • Stock registers |  |  |  |  |
| 6 | • Temperature monitoring records |  |  |  |  |
| 7 | Are the following Truenat TB-related standard operating procedures (SOPs) approved and accessible at the testing site? |  |  |  |  |
| 8 | • Specimen collection |  |  |  |  |
| 9 | • Sample processing for DNA extraction |  |  |  |  |
| 10 | • DNA extraction (Trueprep) |  |  |  |  |
| 11 | • PCR amplification (Truenat micro PCR) |  |  |  |  |
| 12 | • Recording and reporting |  |  |  |  |
| 13 | • Bio-medical Waste management |  |  |  |  |
| 14 | • Spill management |  |  |  |  |
| 15 | Is TruenatSOP/guidance document displayed? |  |  |  |  |
| 16 | Are charts for Do's and Don’t's displayed? Discuss the same with LT |  |  |  |  |
| 17 | Is there evidence that all SOPs, documents and forms have been read by the personnel? |  |  |  |  |
| 18 | Pippeting Volume (6 µl) (Yes /No) |  |  |  |  |
| 19 | Device shifting from One place to another (Yes /No) |  |  |  |  |
| 20 | Sample contaminated (Yes/No), (document %) (*Appropriate Quality & Quantity) |  |  |  |  |
| 21 | High number of paucibacillary load samples (Yes /No)  (document %) |  |  |  |  |
| 22 | Number and Proportion of Errors in Trueprep DNA extraction |  |  |  |  |
| 23 | Number and Proportion of Errors in MTB testing |  |  |  |  |
| 24 | Number and Proportion of MTB Invalid |  |  |  |  |
| 25 | Number and Proportion of MTB/RIF Indeterminate testing |  |  |  |  |
| 26 | Number and Proportion of Errors in MTB/RIF testing |  |  |  |  |
| 27 | Change in Lab staffs or rotation of duties (Yes/No) |  |  |  |  |
| 28 | Are all samples subjected to MTB Detection prior to MTB RIF detection (Yes /No) |  |  |  |  |
| 29 | Proper labelling of the Elute tubes (Yes /No) |  |  |  |  |
| 30 | Delay in testing RIF after MTB confirmation (Yes/No) |  |  |  |  |
| 31 | Ensuring Proper DNA storage (Yes/No) |  |  |  |  |
| 32 | Is dedicated and trained technician available for performing the test? |  |  |  |  |
| 33 | Checking of Washer (Yes/No) |  |  |  |  |
| G | **Quality Assurance** | | | | |
| 1 | Are the essential elements of a quality assurance system in place? |  |  |  |  |
| 2 | • SOPs, training and competence assessment |  |  |  |  |
| 3 | • Lot testing (new batch testing) |  |  |  |  |
| 4 | • Quality indicator monitoring |  |  |  |  |
| 5 | Is the laboratory running negative controls after every 50 tests? |  |  |  |  |
| 6 | Is the laboratory replacing the glass slide after every 200 tests? |  |  |  |  |
| 7 | Are quality indicators being filled and sent to IRL monthly |  |  |  |  |
| 8 | Are pipettes being replaced after every six months |  |  |  |  |
| 9 | Is plastic waste and trunat chips dipped in freshly pepared 1% sodium hypochlorite slolution for 30 minutes before disposal |  |  |  |  |
| 10 | Is the technician trained to manage spills in cartridge holder trays? |  |  |  |  |
| H | **Recording and reporting** | | | | |
| 1 | Is an approved reporting form available to report results of Truenat TB testing? |  |  |  |  |
| 2 | Are laboratory staff familiar with use of the digital flatscreen interface of the Truelab analyzer? |  |  |  |  |
| H | **Monitor and analyze quality indicators (Lab register of Truenat- Last month/quarter data)** | | | | |
| 1 | Are the following quality indicators monitored and analysed by the Truenat TB testing site and reported: |  |  |  |  |
| 2 | • Number and proportion of Truenat TB tests that generated a result of MTBC detected |  |  |  |  |
| 3 | • Number and proportion of Truenat TB tests that generated a result of MTBC not-detected |  |  |  |  |
| 4 | • Number and proportion of Truenat TB tests that generated Invalid results |  |  |  |  |
| 5 | • Number and proportion of Truenat TB tests that generated Error results |  |  |  |  |
| 6 | • Number and proportion of Truenat MTB-RIF Dx tests that generated a result of RIF-resistance detected |  |  |  |  |
| 7 | • Number and proportion of Truenat MTB-RIF Dx tests that generated a result of RIF-resistance not detected |  |  |  |  |
| 8 | • Number and proportion of Truenat MTB-RIF Dx tests that generated In-determinate results |  |  |  |  |
| 9 | • Number and proportion of Truenat MTB-RIF Dx tests that generated Error results |  |  |  |  |
| 10 | How many samples were rejected due to presence of blood, food particles, tobacco, pan masala and leakages before the test is run? |  |  |  |  |
| 11 | • Number and proportion of specimens for which a Truenat TB test result was reported within the target TAT (i.e., time from receipt of specimen to reporting of results) |  |  |  |  |
| J | **Other issues if any:** | | | | |
| 1 | If Error comes what is the practice? |  |  |  |  |
| 2 | If MTB Invalid comes what is the practice? |  |  |  |  |
| 3 | If RIF Indeterminate comes what is the practice? |  |  |  |  |
| 4 | If Power off between run what is the practice? |  |  |  |  |
| 5 | Any blackout/ lab closed, mention reasons. |  |  |  |  |
| 6 | Backlog samples, sample storage mechanism. |  |  |  |  |

Table S1 includes a checklist used during root cause analysis and includes details of Truenat sites and their performance indicator, along with a questionnaire for training and competency assessment; site infrastructure; Equipment, service, and maintenance; supply chain; sample transportation; procedures; quality assurance; recording and reporting; monitor and analyze performance indicators and any other issues pertaining to Truenat MTB-RIF testing.

1.2 Supplementary figures:

Figure S1a: State-wise distribution of Invalid Rates in *Mtb* performing less than 5000 tests per quarter


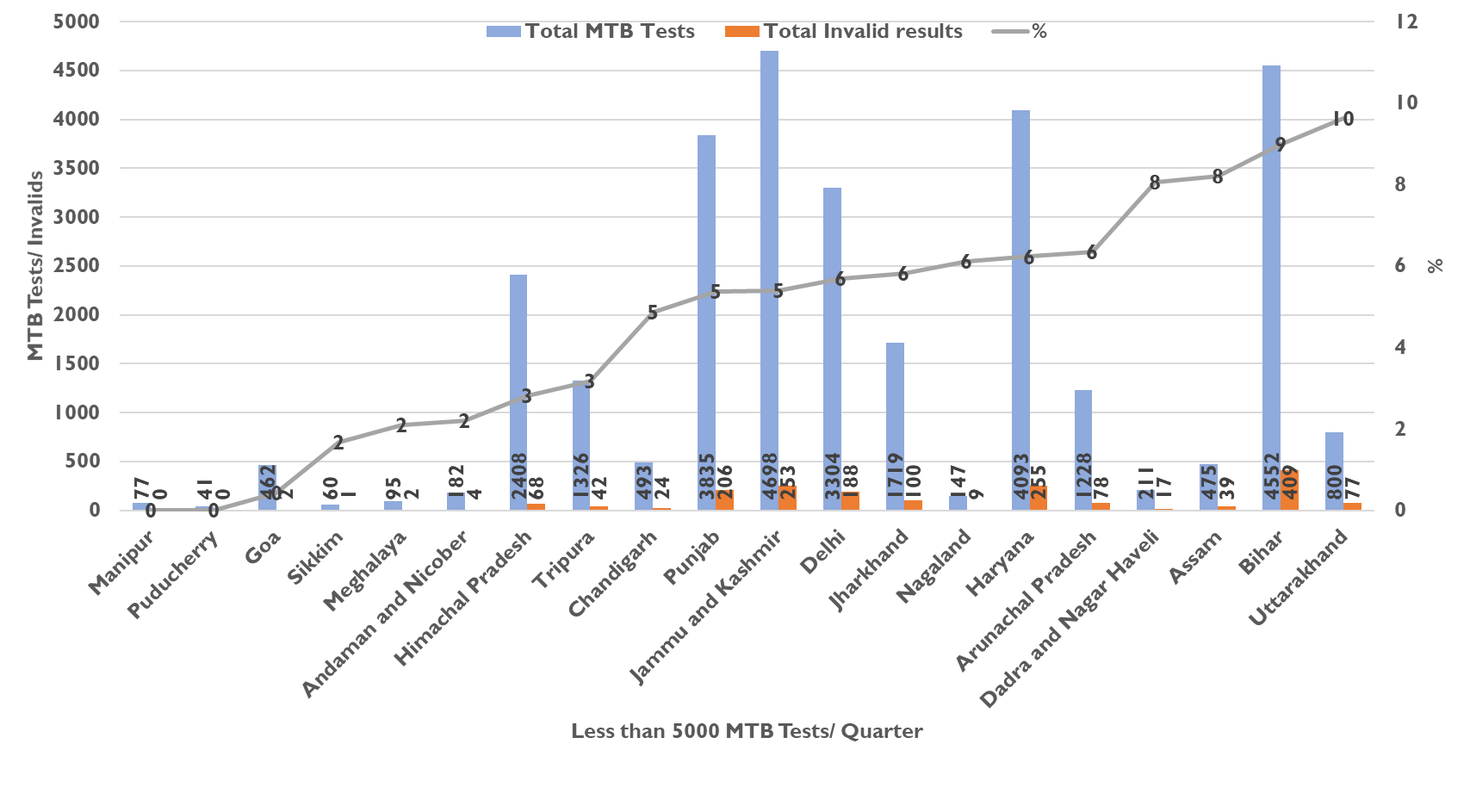


The graph shows state-wise distribution of Invalid Rates in *Mtb* performing less than 5000 tests per quarter. Blue bar represents total MTB tested and orange bar represents the total invalid results. The line graph shows the percentage of invalids among the total MTB tested.

Figure S1b: State-wise distribution of Invalid Rates in *Mtb* performing more than 5000 tests per quarter


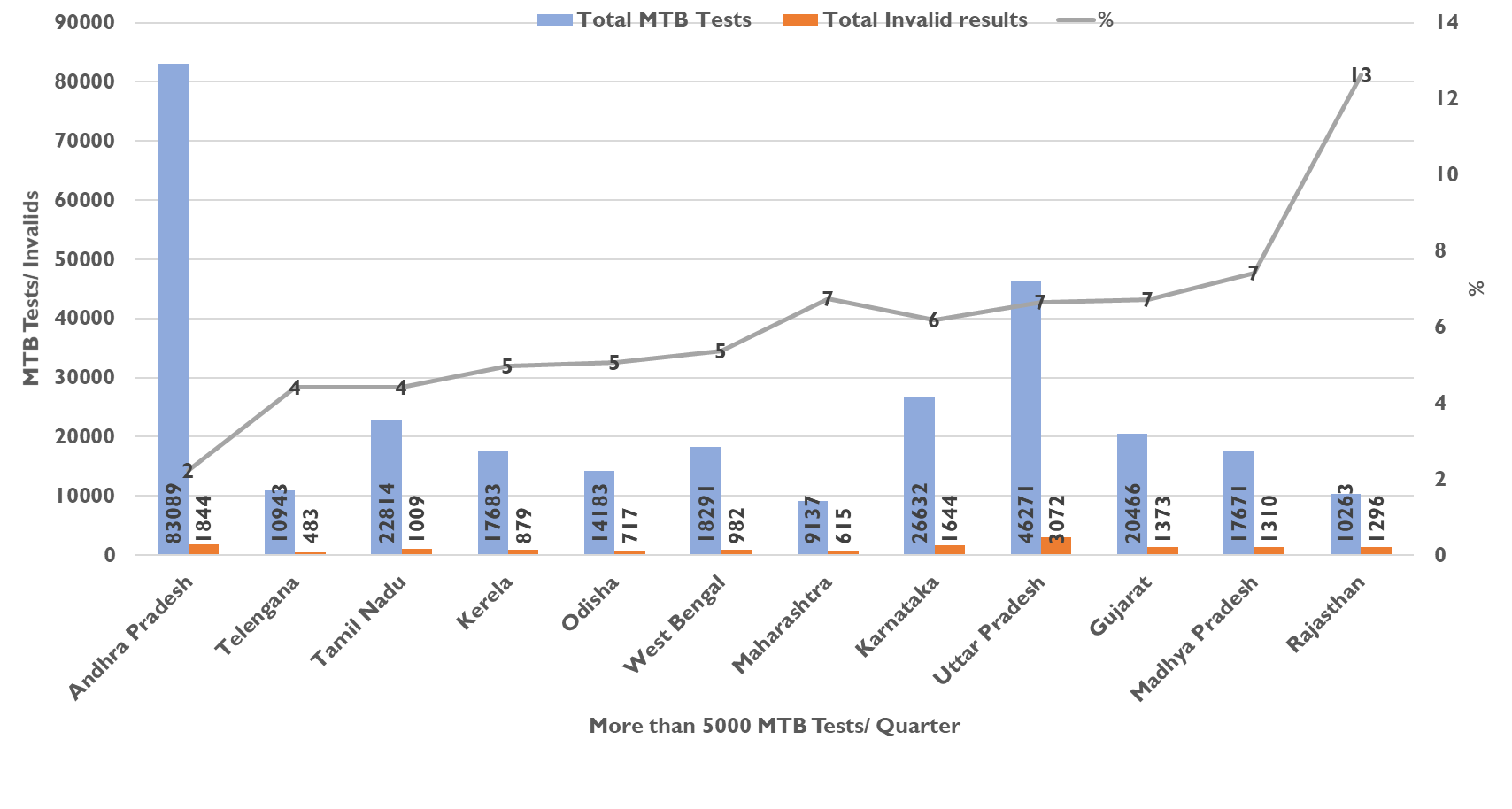


The graph shows state-wise distribution of Invalid Rates in *Mtb* performing more than 5000 tests per quarter. Blue bar represents total MTB tested and orange bar represents the total invalid results. The line graph shows the percentage of invalids among the total MTB tested.

Figure S2a: State wise distribution of Indeterminate Rates in Rifampicin performing less than 1000 tests per quarter


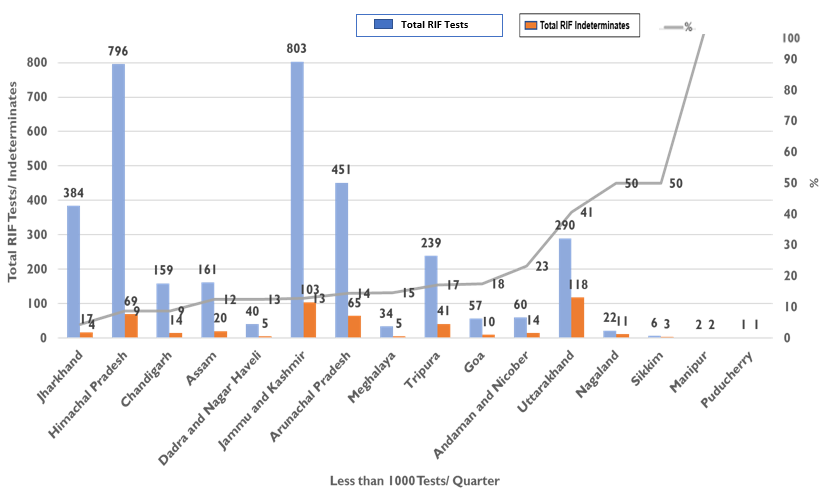


The graph shows state-wise distribution of Indeterminate Rates in Rifampicin testing performing less than 5000 tests per quarter. Blue bar represents total RIF tested and orange bar represents the total indeterminate results. The line graph shows the percentage of indeterminates among the RIF tested.

Figure S2b: State wise distribution of Indeterminate Rates in Rifampicin performing more than 1000 tests per quarter

#
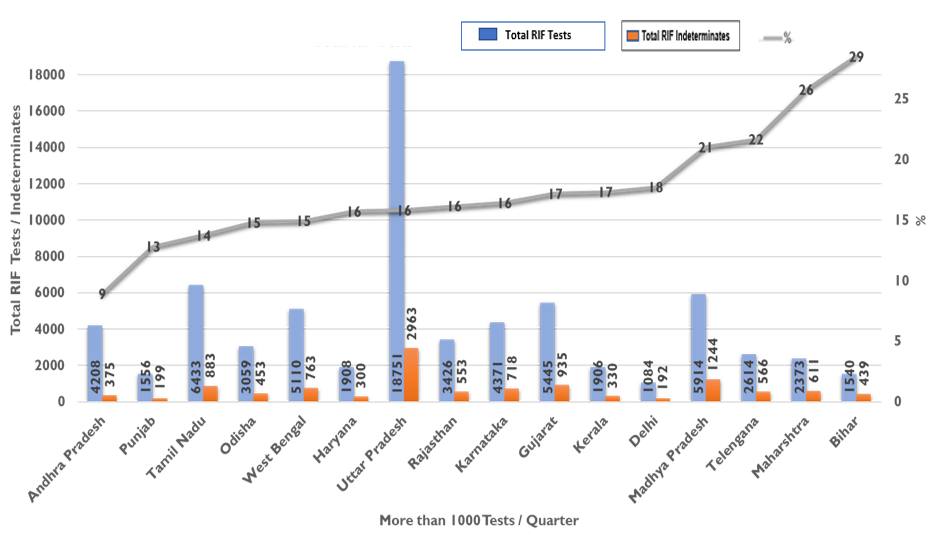


The graph shows state-wise distribution of Indeterminate Rates in Rifampicin testing performing more than 5000 tests per quarter. Blue bar represents total RIF tested and orange bar represents the total indeterminate results. The line graph shows the percentage of indeterminates among the RIF tested.
